# When Postextubation Stridor Is Not Edema: Bilateral Lateral Subglottic Mucosal Tears

**DOI:** 10.7759/cureus.116560

**Published:** 2026-09-19

**Authors:** Alhussein Dharhan, Amreeta V Sharma

**Affiliations:** 1 Medical Intensive Care Unit, Henry Ford St. John Hospital, Detroit, USA; 2 Department of Internal Medicine, St. George’s University School of Medicine, St. George’s, GRD; 3 Department of Pulmonary and Critical Care Medicine, Henry Ford St. John Hospital, Detroit, USA

**Keywords:** airway trauma, endotracheal intubation, iatrogenic tracheal injury, laryngotracheal tear, postextubation stridor, subglottic injury, tracheal laceration, tracheal splinting

## Abstract

Iatrogenic tracheal injury following endotracheal intubation is a rare but potentially life-threatening complication. Most reported injuries involve the posterior membranous tracheal wall. We describe a case in which a patient required rapid sequence emergency intubation in the ED and, three days later, following extubation, developed acute stridor and increasing oxygen requirements, found to be secondary to bilateral, inferiorly based lateral mucosal tears of the subglottic larynx and superior trachea with an associated grade 1 subglottic stenosis. This case highlights the importance of maintaining a high suspicion for airway trauma when a patient presents with difficulty breathing, stridor, or decreasing oxygenation after intubation and supports downsized endotracheal tube splinting as an effective nonsurgical management strategy.

## Introduction

Endotracheal intubation, particularly when performed emergently, carries a recognized risk of airway trauma ranging from minor mucosal abrasion to full-thickness tracheal laceration. The often-cited incidence of approximately one in 20,000 refers to tracheal rupture, and the true incidence is uncertain, with reported estimates varying across publications [1]. Among reported cases of post-intubation tracheal rupture, emergency intubation is the principal risk factor for death, conferring an approximately threefold increase in mortality compared with elective intubation [1]. The classically described injury pattern is a longitudinal tear of the posterior membranous tracheal wall, the noncartilaginous back wall of the trachea, which lacks the structural support of the anterior cartilaginous rings, accompanied by subcutaneous emphysema or pneumomediastinum [1,2]. We report a case of bilateral, laterally based mucosal tears of the subglottic larynx (the region of the larynx immediately below the vocal cords) and proximal trachea following emergent intubation and discuss the diagnostic pitfall of attributing refractory stridor, a high-pitched breath sound indicating upper airway narrowing, to simple laryngeal edema.

## Case presentation

A 64-year-old man with a history of morbid obesity, type 2 diabetes mellitus, end-stage renal disease on hemodialysis, obstructive sleep apnea, and other comorbidities, including coronary artery disease, atrial fibrillation, peripheral arterial disease, heart failure with preserved ejection fraction, hypertension, hyperlipidemia, prior pancreatitis, right above-the-knee amputation, and a right upper extremity arteriovenous fistula, presented to the ED from an assisted living facility with altered mental status of unknown duration. His last known dialysis session was unknown at the time of presentation. Emergency medical services documented hypoxia to 82% on room air, with improvement on nasal cannula oxygen. Vital signs on presentation are summarized in Table 1. On initial examination, the patient was somnolent but oriented to person, place, and time when roused. The patient was in no acute distress, with moist mucous membranes and no meningismus. Work of breathing was easy, although auscultation was limited by body habitus. Cardiac and cranial nerve examinations were within normal limits.

**Table 1:** Vital signs on ED presentation.

Parameter	Value	Reference range
Temperature	98.4 °F (36.9 °C)	97.8-99.1 °F (36.5-37.3 °C)
Blood pressure	145/75 mmHg	<120/80 mmHg
Pulse	90 bpm	60-100 bpm
Respiratory rate	18 breaths/min	12-20 breaths/min
SpO₂ (room air)	82%	95-100%

An initial venous blood gas obtained on arrival demonstrated acute respiratory acidosis with severe hypercapnia and a normal lactate. Concurrent laboratory studies showed leukocytosis (Table 2). Over this interval, the patient’s mental status declined to a Glasgow Coma Scale score of 5, with examination findings consistent with carbon dioxide narcosis and metabolic encephalopathy in the setting of sepsis. He was moved to the resuscitation bay for emergent airway management.

**Table 2:** Initial laboratory values and venous blood gas on arrival.

Parameter	Value	Reference range
pH (venous)	7.1	7.31-7.41
pCO₂ (venous)	78.6 mmHg	41-51 mmHg
pO₂ (venous)	53 mmHg	25-40 mmHg
HCO₃⁻	23 mEq/L	22-26 mEq/L
Lactate	0.8 mmol/L	0.5-2.0 mmol/L
O₂ saturation (venous)	81%	60-80%
White blood cell	19.7 × 10³/µL	4.5-11.0 × 10³/µL
Glasgow Coma Scale	5	15 (normal)

Rapid sequence intubation was performed for altered consciousness and hypercapnic respiratory failure, with an anticipated difficult airway. The patient was unresponsive at the time of the procedure; consent was obtained under the emergency exception. Preoxygenation was performed via a nonrebreather mask. Etomidate and succinylcholine were used for induction and paralysis. Video-assisted laryngoscopy was performed using a Macintosh 3 blade, without bougie assistance, demonstrating a Cormack-Lehane grade 3 view [3]. An 8.0-mm internal diameter (ID) cuffed endotracheal tube, consistent with conventional adult male tube sizing rather than a measured assessment of this patient’s airway caliber, was passed orally under direct visualization through the vocal cords on the first attempt and secured at 24 cm at the lip with a commercial tube holder. Cuff pressure was not recorded with a manometer at the time of emergent intubation. Placement was confirmed by chest rise, colorimetric end-tidal carbon dioxide detection, and chest radiography. The procedure was tolerated well, without immediate complication.

The patient was subsequently found to have sepsis secondary to *Legionella *pneumonia, confirmed by a positive urine antigen test, and was started on intravenous levofloxacin for a seven-day course. He was admitted to the medical intensive care unit and demonstrated clinical improvement.

The patient was extubated on hospital day 3. Within one hour, he developed acute stridor, increased work of breathing, somnolence, and an increasing supplemental oxygen requirement. An arterial blood gas obtained at that time demonstrated acute respiratory acidosis with hypoxemia (Table 3). Symptoms improved partially following administration of dexamethasone and nebulized racemic epinephrine and placement on bilevel positive airway pressure. Otolaryngology was not involved in the initial emergent intubation and was consulted for the first time at this point, following the onset of post-extubation stridor, for bedside airway evaluation.

**Table 3:** Arterial blood gas postextubation (hospital day 3).

Parameter	Value	Reference range
pH (arterial)	7.22	7.35-7.45
pCO₂ (arterial)	57.7 mmHg	35-45 mmHg
pO₂ (arterial)	65.6 mmHg	80-100 mmHg
O₂ saturation (arterial)	86.30%	95-100%

The initial head and neck examination was within normal limits. Bedside flexible laryngoscopy (Video 1) was performed following topical nasal anesthesia. The upper airway structures proximal to the larynx appeared normal, and mild subglottic mucosal edema was the only abnormality noted on this initial pass. Advancing the scope beyond the glottis, however, revealed the underlying injury: paired mucosal flaps disrupting the lateral walls of the subglottic larynx and extending into the uppermost trachea, each tear oriented with its base inferiorly (Figure 1). The underlying cartilage remained structurally intact, and a mild (grade 1) circumferential narrowing was visible just above the tears. The carina was reached without further abnormality.

**Video 1:** Bedside flexible laryngotracheoscopy of the upper airway, subglottic larynx, and proximal trachea.

**Figure 1:**
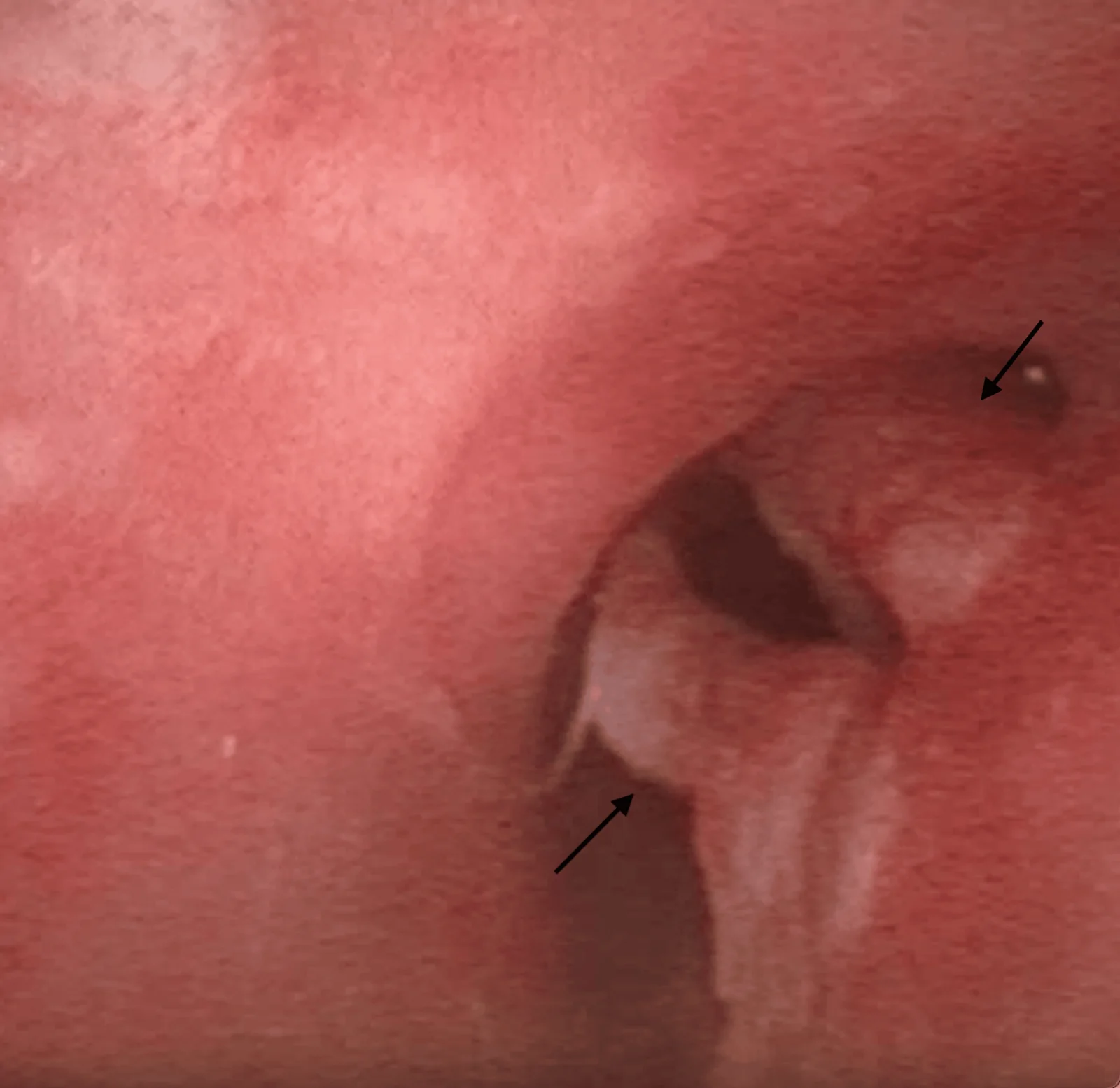
Flexible laryngotracheoscopy demonstrating laterally based mucosal tear and flap (arrows) of the subglottic larynx, visualized during exhalation.

The partially obstructive nature of the defect prompted a cardiothoracic surgery consultation. The airway was secured with a smaller 6.0-mm endotracheal tube, deliberately positioned beyond the injured segment so the tube itself could act as an internal splint. Throughout this period, the patient remained free of palpable neck crepitus and stayed fully saturated on low ventilator support.

A CT of the soft tissues of the neck with and without contrast (Figure 2) and a CT of the chest (Figure 3) were obtained the following day, showing no evidence of thoracic trauma, emphysema, or acute process in the neck. Esophagogastroduodenoscopy performed the next day showed no acute findings, excluding esophageal injury. The patient was managed with dexamethasone 10 mg every eight hours, twice-daily proton pump inhibitor therapy, and strict glycemic control, with a plan for trial extubation at 72 hours.

**Figure 2:**
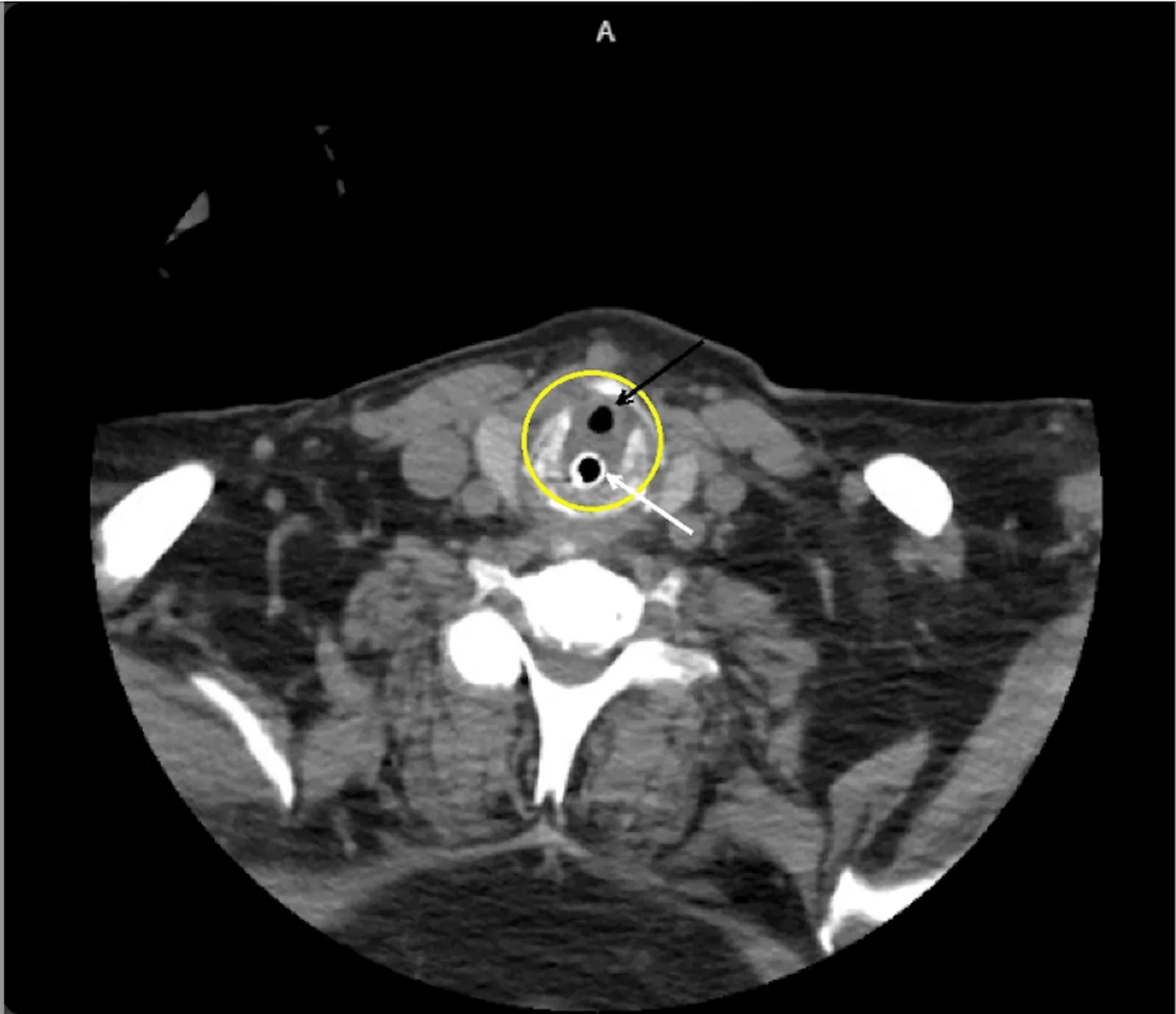
CT of the neck soft tissues at the level of the subglottic larynx, soft tissue window. The yellow circle denotes the tracheal air lumen (black arrow), immediately adjacent to a nasogastric feeding tube within the esophageal lumen (white arrow). No radiographic evidence of tracheal wall discontinuity is identified at this level.

**Figure 3:**
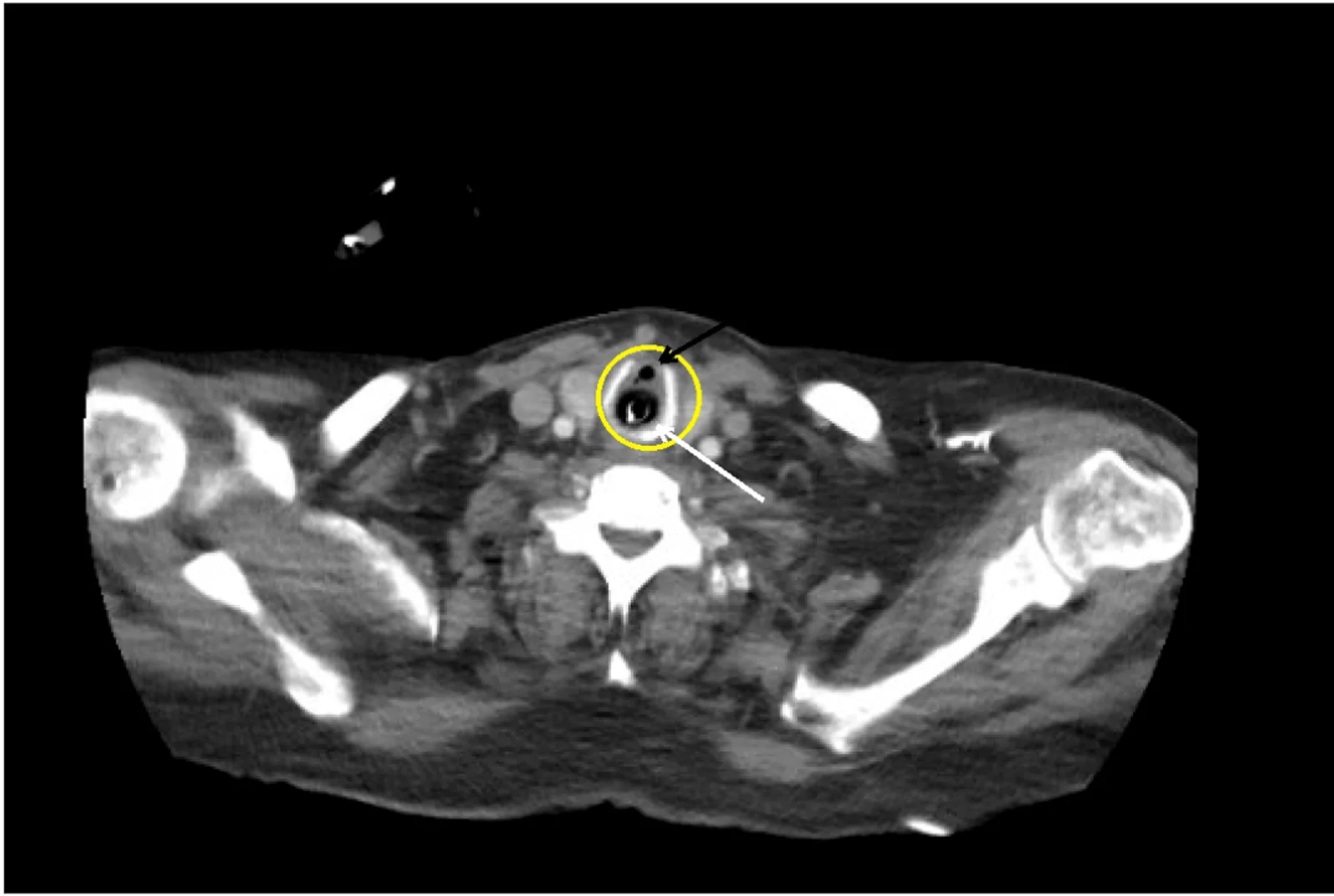
CT of the chest at the level of the thoracic inlet, soft tissue window. The yellow circle denotes the tracheal air lumen (black arrow) at an adjacent level, again immediately adjacent to the nasogastric feeding tube within the esophageal lumen (white arrow). No radiographic evidence of tracheal wall discontinuity is identified, nor are there signs of emphysema or thoracic cavity trauma.

Extubation was reattempted 72 hours later and went smoothly. A follow-up scope showed near-complete resolution of the earlier findings. The lateral flaps had re-epithelialized, the cartilage remained undamaged, and the mild subglottic narrowing seen previously was no longer present. Otolaryngology closed out their involvement at that point, and the patient was discharged on a month of acid suppression and inhaled corticosteroid therapy to discourage granulation tissue.

The patient’s subsequent hospital course was uneventful; he was transferred out of the intensive care unit to a dialysis-capable floor and was discharged on hospital day 9 to a subacute rehabilitation facility.

## Discussion

Airway trauma during intubation exists on a spectrum, and the mechanisms producing the classic posterior membranous tear, including cuff overinflation, direct trauma from the stylet or tube tip, and shear forces during difficult or repeated laryngoscopy, are well described [1,2]. What is less appreciated is how often such injury goes unrecognized in real time. The endotracheal tube, its inflated cuff, and the effects of positive-pressure ventilation can all mechanically splint a damaged airway wall, suppressing the signs that would otherwise prompt evaluation [1,4]. As a result, many injuries are first identified only after extubation, when loss of that splinting effect allows previously silent trauma to declare itself as stridor, respiratory distress, or hypoxia [4]. Subcutaneous emphysema and pneumomediastinum are the most widely recognized presenting signs of a full-thickness injury, and their absence has been associated with delayed diagnosis and, in cases of full-thickness disruption, increased mortality [1].

Rather than involving the posterior membranous wall as classically described [1,2], this injury was confined to the lateral subglottic and proximal tracheal mucosa, sparing the posterior and cartilaginous structures entirely. To contextualize this pattern, a PubMed search was performed on September 16, 2026, using the terms (“tracheal rupture” OR “tracheal laceration” OR “tracheal tear” OR “subglottic”) AND (“intubation” OR “post-intubation” OR “iatrogenic”), which yielded 148 records. Titles and abstracts were screened, with full texts retrieved where the injury pattern was unclear from the abstract, including non-English reports (Spanish and German). No case describing bilateral, laterally based mucosal tears of the subglottic larynx and proximal trachea following endotracheal intubation was identified; reported injuries were predominantly posterior membranous, full-thickness, or surgically inflicted. To our knowledge, this bilateral, laterally based subglottic pattern has not been previously reported.

The differential diagnosis of postextubation stridor is broad. The most common cause is laryngeal edema, which results from mechanical mucosal trauma during intubation and is the most frequent cause of postextubation upper airway obstruction [5]. Other causes include laryngospasm, provoked by reflexogenic stimulation of an already inflamed larynx, and retained secretions with a weak or ineffective cough [5]. Structural laryngeal lesions, including mucosal ulceration, granulation tissue, and abnormal vocal fold mobility, are less common but important considerations, particularly when the response to standard therapy is incomplete [6]. Because laryngeal edema accounts for the majority of cases and this patient carried several recognized risk factors, including emergent intubation, an anticipated difficult airway, a relatively large (8.0-mm ID) endotracheal tube, and obesity, it was a reasonable initial presumption, and treatment was appropriately directed at edema with corticosteroids and nebulized epinephrine [4,5].

This patient also lacked subcutaneous emphysema throughout his course [1]. The absence of crepitus here likely reflected the partial-thickness, mucosal nature of the injury rather than a full-thickness tracheal breach, which was excluded on both direct visualization and CT.

Management was entirely conservative. A deliberately downsized endotracheal tube was selected to minimize further mucosal contact trauma at the injured segment while still providing a mechanical scaffold, and it was positioned with its tip and cuff distal to the tears so the tube itself could splint the lacerated mucosa and shield it from positive-pressure airflow during healing. This was combined with corticosteroids and acid suppression to limit granulation tissue formation. This mirrors conservative strategies described for other post-intubation tracheal injuries, including tears of greater depth and length than seen here [1,7]. Repeat endoscopy four days later confirmed complete healing and resolution of the subglottic stenosis, supporting downsized tube splinting as effective for partial-thickness, noncartilaginous injuries, obviating surgical repair.

This patient’s comorbidities, including morbid obesity and diabetes mellitus, may be relevant to both his airway complication risk and healing capacity, although causality cannot be established from a single case [8]. Otolaryngology involvement occurred only reactively after the development of postextubation stridor rather than at the time of initial airway management.

This report has several limitations. As a single case, it cannot establish incidence, causality, or generalizability. Documentation of the initial intubation was incomplete: cuff pressure was not measured with a manometer, and exact tube tip depth was not recorded, precluding assessment of cuff overinflation or tube position as contributing mechanisms. Pre-intubation flexible laryngoscopy was not performed, as the intubation was emergent and the patient was unresponsive, so airway caliber before tube placement could not be assessed. Endoscopic follow-up confirmed mucosal healing in the short term, but no long-term airway surveillance was obtained, leaving the durability of the result and the possibility of late stenosis unknown. Finally, only one intubation attempt with a single Cormack-Lehane grade 3 view was documented, and the mechanism producing a bilateral, laterally based rather than posterior membranous injury could not be established from the available record.

## Conclusions

This case illustrates that post-intubation tracheal injury can present subtly as refractory postextubation stridor, mimicking simple laryngeal edema, and may take the form of bilateral, laterally based subglottic mucosal tears rather than the classic posterior membranous wall laceration. In this patient, postextubation stridor that responded only partially to corticosteroids and racemic epinephrine, despite the absence of subcutaneous emphysema, was ultimately explained by a structural mucosal injury identified on direct airway visualization, suggesting that direct airway examination may be worth considering when postextubation stridor is refractory to standard therapy. Conservative management with a downsized endotracheal tube for internal splinting, corticosteroids, and acid suppression was associated with complete mucosal healing without surgical intervention in this case, although generalizability is limited by the single-case nature of this report.
